# Portal vein resection in advanced pancreatic adenocarcinoma: is it worth the risk?

**DOI:** 10.1007/s00508-016-1024-7

**Published:** 2016-06-30

**Authors:** Katharina Marsoner, Rainer Langeder, Dora Csengeri, Gottfried Sodeck, Hans Jörg Mischinger, Peter Kornprat

**Affiliations:** 1 Department of General Surgery, Medical University of Graz, Auenbruggerplatz 29, 8036 Graz, Austria; 2 Department of Emergency Medicine, Medical University of Vienna, Vienna, Austria

**Keywords:** Pancreatic ductal adenocarcinoma, Pancreaticoduodenectomy, Portal vein resection, Perioperative outcome, Long-term survival

## Abstract

**Introduction:**

Portal vein resection represents a viable add-on option in standard pancreaticoduodenectomy for locally advanced ductal pancreatic adenocarcinoma, but is often underused as it may set patients at additional risk for perioperative and postoperative morbidity and mortality. We aimed to review our long-term experience to determine the additive value of this intervention for locally advanced pancreatic adenocarcinoma.

**Patients and methods:**

Single, university surgical center audit over a 13-year period; cohort comprised 221 consecutive patients undergoing pancreatic resection; in 47 (21 %) including portal vein resection. Predictors for short- and long-term survival were assessed via multivariate logistic and Cox regression.

**Results:**

Baseline and perioperative characteristics were similar between the two groups. However, overall skin-to-skin times, intraoperative transfusion requirements as the need for medical inotropic support were higher in patients undergoing additional portal vein resection (*p < *0.0001; *p = *0.001 and *p = *0.03). Postoperative complication rates were 34 vs. 35 % (*p = *0.89), 14 patients (5 % vs. 11 %; *p = *0.18) died in-hospital. An American Society of Anesthesiologists Score >2 was the only independent predictor for in-hospital mortality (OR 10.66, 95 % CI 1.24–91.30). Follow-up was complete in 99.5 %, one-year survival was 59 % vs. 70 % and five-year overall survival 15 % vs. 12 % with and without portal vein resection, respectively (Log rank: *p = *0.25). For long-term outcome, microvascular invasion (HR 2.03, 95 % CI 1.10–3.76) and preoperative weight loss (HR 2.17, 95 % CI 1.31–3.58) were independent predictors.

**Conclusion:**

Despite locally advanced disease, patients who underwent portal vein resection had no worse perioperative and overall survival than patients with lower staging and standard pancreaticoduodenectomy only. Therefore, the feasibility of portal vein resection should be evaluated in every potential candidate at risk.

## Introduction

Complete surgical resection of pancreatic ductal adenocarcinoma (PDAC) represents the key factor for survival despite advances in chemo- and radiochemotherapy. In the 1970s, Fortner first described a radical en bloc surgical resection of venous portal branches and surrounding tissues [1]. Despite this approach might improve survival in locally advanced PDAC, surgeons often are concerned about this technique in fear of the potential additional risk for perioperative and postoperative morbidity and mortality [2–5]. We aimed therefore to review our long-term experience to determine the additional value of portal venous resection in locally advanced PDAC.

## Materials and methods

### Study procedures

Single center, university surgical center audit over a 13-year period; cohort compromised 221 consecutive patients (112 females; median age 67 years [IQR interquartile range(60–72)]) undergoing pancreatic resection for diagnosis of PDAC; in 47 (21 %) portal vein resection (PVR) has been performed as an add-on due to locally advanced disease making a R0 resection impossible without additional resection of portal venous tissue. Baseline and perioperative risk factors were recorded; the ASA (American Association of Anesthesiologist’s) Score was applied to estimate the perioperative risk [6]. All eligible patients underwent a standardized preoperative screening program including detailed physical examination including tumor markers carcino embryotic antigen (CEA), CA 19-9 (carbohydrate antigen) and liver function tests; preoperative oncological staging comprised positron emission tomography (PET) scan, endoscopic retrograde cholangiopancreaticography (ERCP) and/or magnetic resonance cholangiopancreaticography (MRCP) and esophagogastroduodenoscopy (EGD). If not already performed at initial diagnosis, all patients received a multidetector row computed tomography (MDCT) with pancreas protocol. Prior to definite decision for surgery all patients were admitted to the institutional tumor board including surgeons, oncologists, histopathologists, and radiologists. The institutional review board approved the study and waived the need for patient consent.

### Portal vein resection technique

Depending on the primary tumor location, either a standard or pylorus-preserving pancreaticoduodenectomy, distal pancreatectomy with splenectomy or total pancreatectomy was carried out in all cases. The biliodigestive anastomoses were connected with 5–0 or 6–0 double layer single sutures; the biliodigestive anastomoses were performed routinely via end-to-side pancreaticojejunostomy and hepaticojejunostomy and were protected by means of internal drainages.

Portal vein resection was performed in all cases of radiologic and/or intraoperative diagnosis of tumorous adherence or infiltration of the portal venous wall, allowing a potential R0 resection with additional resection of the portal venous segment. Prior to cross-clamp of the portal venous branches all patients received intravenously 5000 international units (IU) heparin. In case that tumor infiltration of the venous wall was less than one-third of the circumference, this particular segment was resected in an elliptic fashion and reconstructed with a Gore-tex® patch; otherwise the affected vein segment was resected completely: reconstruction of the portal axis was achieved via a tension-free end-to-end anastomosis with a continuous running 6–0 polypropylene suture or via a Gore-tex® tube graft when a longer vein segment had to be resected. Heparinization was not reversed at the end of the procedure and continued intravenously until oral prescription of unlimited aspirin or clopidogrel upon discretion of the surgeon. Intraabdominal drainages were placed routinely in all patients before closure of the abdominal wall.

### Histopathological examination

Intraoperative rapid frozen section diagnosis was part of the standard protocol and according to the intraoperative histopathologic diagnosis, resection was extended until negative margins could be obtained or the surgical strategy was switched to a total pancreatectomy. In definitive histopathological examination, a hematoxylin–eosin staining according to standard procedures was performed, in cases with portal venous involvement, the grade of vascular infiltration was differentiated between adhesion, adventitial, media, or transmural infiltration.

### Follow-up protocol

Postoperative morbidity was classified according to Clavien and Dindo (CDC) [7]. A drainage cholangiography was performed in all patients before discharge to exclude anastomotic leakage. Patients underwent clinical, laboratory, and radiological (MDCT) follow-up 3, 6, and 12 months postoperatively. Long-term mortality data were obtained from the Austrian National Cancer registry [8].

### Statistical analysis

If not otherwise indicated, continuous variables were reported as median and interquartile range; categorical data were reported as count and percentages. Categorical variables were compared with Fisher’s exact text or the χ^2^ test, as appropriate; metric variables were compared with the Wilcoxon test. A multivariate logistic regression model was applied to assess the strongest independent risk factor for intrahospital mortality. Results of the statistic regression model are given as the odds ratio (OR) and 95 % confidence interval (CI). A resampling model with 1000 replications was then used to confirm CI. Regression diagnostics and overall model fit were performed according to standard procedures, providing Hosmer–Lemeshow tests for calibration and C statistics for discrimination of the final model. Overall survival and progression-free survival were calculated according to the method of Kaplan and Meier. A Cox regression model was used for multivariate and univariate analysis to identify predictors for worse long-term outcome. A two-sided *p*-value <0.05 was considered statistically significant and SPSS 22.0 for Windows (IBM Inc, Somers, NY, USA) was used for all statistical analyses.

## Results

Over a 13-year period, 221 consecutive patients (112 females; median age 67 years [IQR 60–72]) were subject to pancreatic resection for diagnosis of PDAC at our institution; in 47 (21 %) portal vein resection was deemed necessary as an add-on due to locally advanced disease.

Detailed demographics and cohort comparisons are given in Tables 1 and 2: Baseline and perioperative characteristics were similar between the two groups without statistical significance except preoperative finding of portal venous infiltration in radiological imaging (*p* < 0.0001) Obviously due to the additional procedure, overall skin-to skin times (median 361 [IQR 311–422] vs. 307 [IQR 236–361] minutes in patient with and without PVR, respectively), transfusion requirements (85 vs. 55 % of patients) as the need for medical inotropic support (32 vs. 16 %) were higher in patients undergoing additional portal vein resection (*p < *0.0001; *p = *0.001 and *p = *0.03). Detailed histopathological results were given in Tables 3 and 4.

**Table 1:** Baseline and preoperative demographic patient’s characteristics

Characteristics	Overall (*n = *221)	Without portal vein resection (*n = *174)	With portal vein resection (*n = *47)	Two-sided *p* value
*Demographic data*				
Age (years)	67 (60–72)	67 (60–73)	66 (60–71)	0.25
Female sex	112 (51 %)	89 (51 %)	23 (49 %)	0.87
BMI	24 (22–28)	25 (22–28)	23 (22–26)	0.09
Age >70 years	75 (34 %)	62 (36 %)	13 (27 %)	0.39
*Chronic health conditions (%)*				
Alcohol abuse	19 (9)	14 (8)	5 (11)	0.57
Nicotine abuse	41 (19)	32 (19)	9 (19)	1.00
Chronic pancreatitis	27 (13)	22 (13)	5 (11)	1.00
Diabetes	60 (27)	48 (28)	12 (26)	0.86
Liver/renal cysts	9 (4)	7 (4)	2 (4)	1.00
ASA >2	133 (62)	109 (64)	24 (52)	0.17
Gastrointestinal comorbidity	43 (19)	33 (19)	10 (21)	0.68
Cardiovascular comorbidity	132 (60)	108 (62)	24 (51)	0.18
Pulmonary comorbidity	27 (12)	24 (14)	3 (6)	0.21
Other comorbidities	71 (34)	56 (34)	15 (34)	1.00
Extrapancreatic malignancy (current and/or anamnestic)	36 (16)	28 (16)	8 (17)	1.00
*Preoperative symptoms (%)*				
Emesis	22 (10)	17 (10)	5 (12)	0.78
Abdominal pain	107 (50)	83 (49)	24 (56)	0.50
Jaundice	64 (30)	50 (29)	14 (32)	0.72
Acute pancreatitis	27 (13)	20 (12)	7 (16)	0.44
Diarrhea	16 (8)	13 (8)	3 (7)	1.00
Weight loss	66 (30)	51 (30)	15 (32)	0.86
*Preoperative diagnostics (%)*				
CT	176 (84)	137 (84)	39 (83)	0.83
MRI	122(59)	89 (55)	33 (70)	0.09
PET-CT	65 (31)	45 (28)	20 (46)	0.07
ERCP	85 (40)	63 (38)	22 (47)	0.31
PTCD	17 (8)	14 (8)	3 (6)	1.00
*Radiol. tumor size (%)*				
<3 cm	82 (49)	61 (35)	21 (52)	0.23
3–5 cm	59 (36)	42 (33)	17 (29)	0.10
5–7 cm	17 (10)	16 (13)	1 (3)	0.08
>7 cm	6 (4)	5 (4)	1 (3)	0.21
Not available	63 (29)			
Biopsy preop	25 (12)	18 (11)	7 (15)	0.45
Radiological portal venous infiltration	32 (14)	13 (7)	19 (47)	<0.0001

*BMI* Body mass index, *ASA Score* Risk Score of the American Society of Anesthesiologists, *CT* Computed tomography, *MRI* Magnetic resonance imaging, *PET-CT* positron emission tomography, *ERCP* endoscopic retrograde cholangiopancreaticography, *PTCD* percutaneous transhepatic cholangio-drainage

**Table 2:** Intra- and postoperative patient’s characteristics

Characteristics	Overall (*n = *221)	Without portal vein resection (*n = *174)	With portal vein resection (*n = *47)	Two-sided *p* value
*Intraoperative data*				
Skin-to-skin time (min)	318 (248–375)	307 (236–361)	361 (311–422)	<0.0001
Kausch Whipple (%)	77 (35)	58 (33)	19 (40)	0.37
Pylorus preserving pancreaticoduodenectomy (%)	65 (30)	54 (31)	11 (23)	0.31
Central resection (%)	1 (1)	1 (1)	0	0.60
Distal pancreatectomy (%)	47 (21)	40 (23)	7 (15)	0.23
Total pancreatectomy (%)	31 (14)	21 (12)	10 (21)	0.11
Splenectomy (%)	68 (32)	51 (29)	17 (36)	0.48
Internal drainage (%)	122 (58)	94 (54)	28 (60)	0.74
Intraoperative blood transfusion (%)	108 (62)	74 (55)	34 (85)	0.001
Intraoperative inotropic support (%)	37 (19)	23 (16)	14 (32)	0.03
*Postoperative patient’s characteristics*				
ICU stay (days)	3 (2–5)	3 (2–4.5)	3 (2–5)	0.11
In-hospital stay (days)	24 (18–31)	23 (17–31)	25 (20–31)	0.23
Postoperative blood transfusion (%)	34 (18)	9 (5)	5 (11)	0.18
In-hospital mortality (%)	14 (6)	9 (5)	5 (11)	0.18

unless otherwise indicated, data are number (percentage),* IQR* interquartile range, * ICU* intensive care unit

**Table 3:** Histopathological details

Characteristics	Overall (*n = *221)	Without portal vein resection (*n = *174)	With portal vein resection (*n = *47)	Two-sided *p* value
*Histopathological data*				
Pancreatitis (%)	62 (30)	51 (32)	11 (24)	0.36
Perineural invasion (%)	49 (23)	36 (21)	13 (28)	0.32
Lymphovascular invasion (%)	105 (52)	79 (50)	26 (59)	0.31
R0 [dorsal margin <1 mm (%)]	106 (60)	87 (62)	19 (50)	0.24
R0 (%)	180 (87 %)	141 (87 %)	39 (89)	0.76
Lymph node ratio	0.16 (0.06–0.33)	0.16 (430.05–0.33)	0.13 (0.07–0.29)	0.43

**Table 4:** Details of portal vein infiltration

Depth of portal vein infiltration	*n* (%)
Adventitia	6 (13)
Media	5 (11)
Transmural	11 (23)
Tumor adherence	15 (32)
No specification	10 (21)

Postoperative complication rates according to Clavien and Dindo (CDC) were 34 vs. 35 % (*p = *0.89), and 14 patients (5 vs. 11 %; *p = *0.18) died in-hospital. An ASA Score >2 was the only independent predictor for in-hospital mortality in logistic regression analysis (OR 10.66, 95 % CI 1.24–91.30; H/L = 0.73, c‑statistics: *p = *0.79). The strong influence of an ASA Score >2 on in-hospital mortality could be confirmed in bootstrap analysis (95 % CI 1.04–22.12; Table 5).

**Table 5:** Results of univariate logistic regression analysis of in-hospital mortality concerning selected potential predictors’

Risk factor	OR* (95 % CI**)	Two-sided *p*-value
Sex	0.35 (0.08–1.53)	0.16
Age >70 years	1.43 (0.36–5.68)	0.61
Alcohol abuse	1.34 (0.21–8.49)	0.76
Diabetes mellitus	0.26 (0.05–1.47)	0.13
*ASA Score> II*	*10.66 (1.24–91.30)*	*0.03*
Preop. weight loss	0.80 (0.18–3.52)	0.77
Preop. jaundice	1.08 (0.25–4.66)	0.92
Preop. pancreatitis	1.05 (0.17–6.37)	0.96
ERCP preop	0.67 (0.16–2.79)	0.58
UICC > II	3.90 (0.58–26.25)	016
PVR	2.31 (0.57–9.34)	0.24

*OR* Odds ratio, *CI* Confidence interval, *ASA Score* Anesthesiologic risk score of the American society of anesthesiology, *ERCP* Endoscopic retrograde cholangiopancreaticography, *UICC* Union Internationale contre la Cancer; clinical tumor stage, PVR portal vein resection

**Fig. 1:**
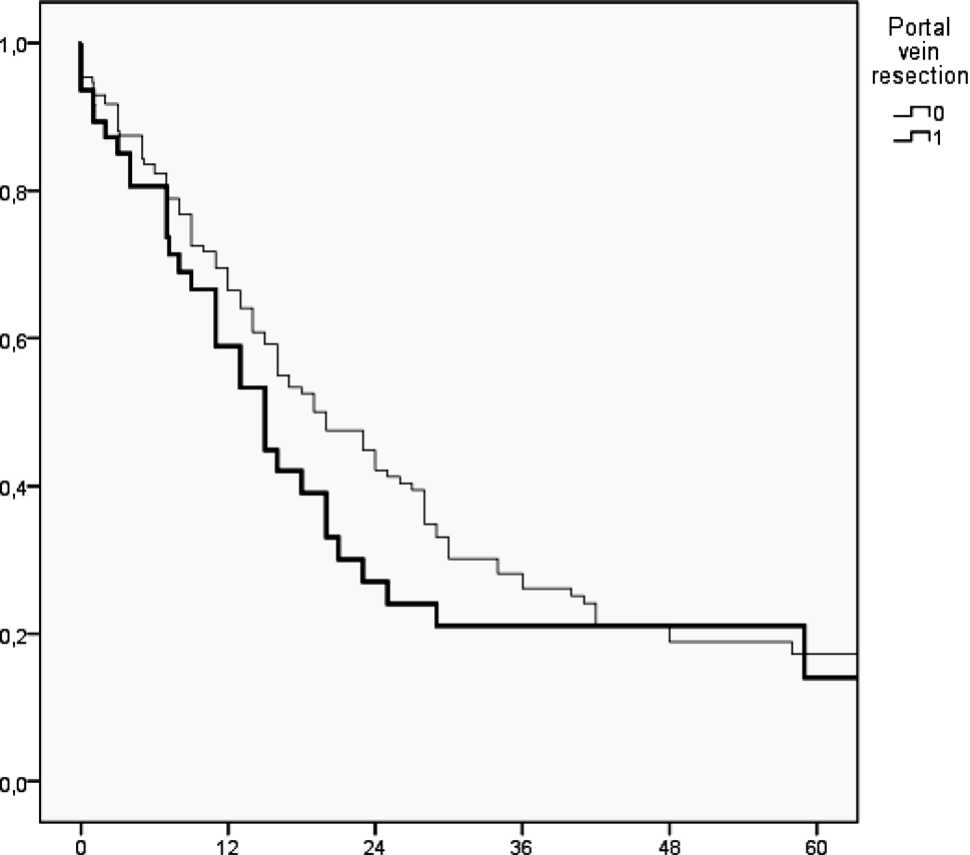
Kaplan–Meier plot of overall survival in patients with and without portal vein resection’

Follow-up was complete in 99.5 % and comprised 4393 patients–months of follow-up (for details Table 6): 1‑year and 5‑year overall survival were 59 and 15 % vs. 70 and 12 % with and without PVR, respectively (Log-rank: *p* = 0.25; Fig. 1).

**Table 6:** Comparison of patients with and without portal vein resection by follow-up characteristics

Follow up characteristics	Overall cohort	Without portal vein resection (*n = *174)	With portal vein resection (*n = *47)	Two-sided *p*-value
Follow-up (months)	12 ( 6–26)	12 (6–28)	11 (7–20)	0.52
*Status at follow up (%)*				
NED	46 (21)	36 (21)	10 (21)	0.93
AWD	31 (14)	27 (16)	4 (9)	0.22
DOD	140 (63)	107 (48)	33 ()	0.27
DOC	3 (1)			
Lost of follow-up	1 (0.5)			
Chemotherapy	126 (57)	98 (56)	27 (60)	0.94
Radiotherapy	3 (1)	2 (1)	1 (2)	0.61
Progression-free survival (months)	7 (4–16)	8 (4–15)	7 (4–16)	0.43

*n* = 2: bronchial carcinoma, *n* = 1: pneumonia; unless otherwise indicated, data are number (percentage);* IQR* interquartile range,* NED* No evidence of disease, *AWD* Alive with disease, *DOD* Dead of disease, *DOC* Dead of other cause

For mid-term and long-term outcome, microvascular invasion (hazard ratio, HR 2.03, 95 % CI 1.10–3.76), preoperative weight loss (HR 2.17, 95 % CI 1.31–3.58), lymphovascular invasion (HR 1.59, 95 % CI 1.03–2.45) and tumor grading (HR 1.78 95 % CI 1.23–2.57) were independent predictors for mortality during follow-up; but not portal vein resection (HR 0.61, 95 % CI 0.33–1.15); Whipple procedure (HR 0.48 95 % CI 0.01–0.75) and chemotherapy (HR 0.05 95 % CI 0.01–0.27) were associated with lower risk of death during follow-up (Table 7).

**Table 7:** Uni- and multivariate predictors for survival (Cox regression)

Predictor	Univariate (HR/95 % CI)	Multivariate (HR/95 % CI)
Sex	0.82 (95 CI 0.60–1.14)	1.09 (95 CI 0.71–1.68)
Age >70 years	1.31 (95 CI 0.92–1.86)	1.39 (95 CI 0.87–2.22)
Diabetes mellitus	1.01 (95 CI 0.75–1.56)	1.66 (95 CI 1.04–2.63)
Cardiovascular comorbidity	0.89 (95 CI 0.63–1.24)	0.62 (95 CI 0.39–0.99)
Preoperative symptomatic	1.25 (95 CI 0.85–1.82)	0.93 (95 CI 0.59–1.48)
Preoperative weight loss	1.45 (95 CI 1.00–2.09)	2.17 (95 CI 1.31–3.58)
Whipple procedure	0.56 (95 CI 0.39–0.79)	0.48 (95 CI 0.31–0.75)
Portal vein resection	1.18 (95 CI 0.79–1.76)	0.61 (95 CI 0.33–1.15)
Free margin (R0)	0.90 (95 CI 0.62–1.13)	0.78 (95 CI 0.50–1.22)
Vascular invasion	1.62 (95 CI 1.12–2.35)	2.03 (95 CI 1.10–3.76)
Lymphovascular invasion	1.63 (95 CI 1.15–2.32)	1.59 (95 CI 1.02–2.45)
Perineural invasion	1.52 (95 CI 1.02–2.24)	1.53 (95 CI 0.95–2.47)
Organ infiltration	1.70 (95 CI 1.17–2.47)	1.04 (95 CI 0.63–1.71)
Tumor grading	1.79 (95 CI 1.35–2.37)	1.78 (95 CI 1.23–3.57)
Chemotherapy	0.16 (95 CI 0.05–0.53)	0.05 (95 CI 0.01–0.27)

*CI* Confidence interval, *HR* hazard ratio

## Discussion

As to our institutional long-term experience portal vein resection represents a viable add-on option in pancreatic surgery for locally advanced PDAC: compared to patients without locally advanced disease, short- and long-term results are acceptable without significant differences.

In recent decades, infiltration of portal venous and/or mesenteric venous branches has ceased to be synonymous with inoperability [1, 9]. Portal vein resection represents therefore the last option for patients with locally advanced disease to receive potential curative surgery [1–5, 10–12] after diagnosis of PDAC.

However, there is still an ongoing debate if pancreatic surgery with vascular resection and reconstruction increases the incidence of postoperative complications and morbidity [2–5, 9–12]. Our postoperative complication rates according to Clavien and Dindo (CDC) were 34 vs. 35 % (*p* = 0.89), and 14 patients (5 vs. 11 %; *p* = 0.18) died in-hospital, which are in line with previously reported data for stand-alone pancreaticoduodenectomy [13, 14]. This particular finding might be explained by the fact that our hepatobiliary surgeons undergo extensive training in the vascular surgical techniques. As a result, only two patients required revision for bleeding; in one patient it was probably due to development of a pancreatic fistula with subsequent erosion hemorrhage; in the remaining patient, the bleeding occurred within 24 h postoperatively.

Further in contrast to others, all patients receive continuous intravenous heparin in the early postoperative period followed by platelet inhibitors in the late postoperative period [15]. Indeed, only one patient experienced portal vein thrombosis together with abscess formation a few days after operation and an unfavorable outcome. Computed tomography follow-up has proven the patency of portal venous reconstructions in all discharged patients.

Current evidence is mixed regarding the risk of the surgical procedure itself and postoperative morbidity and mortality; although numerous publications propose different scoring systems to predict perioperative mortality and morbidity for pancreatic resection, no clinically established preoperative scoring system is available at this point [16, 17]. As to our observation, portal vein resection has no impact upon immediate survival; an ASA Score >2 was the only independent predictor for in-hospital mortality in logistic regression analysis (OR 10.66, 95 % CI 1.24–91.30) has confirmed via bootstrap modelling. Indeed, all patients but one who died during their intrahospital stay were scored into ASA class 3 or 4. As to our opinion, this well-established anesthesiologic score gives an excellent picture of patient’s preoperative general health conditions not only predicting the anesthesiologic risk alone but also the perioperative risk for pancreatic resections.

Median overall survival in our cohort did not differ between the cohorts with and without additional portal vein resection. This finding is in line with other publications in the field and would suggest that this intervention is not worth the effort for locally advanced PDAC [10–12, 18]. However, from the oncologic point of view, it is the only chance for patients with locally advanced PDAC to achieve similar survival rates compared to those patients with lower staging [10–12, 18]. As to our observation, besides extensive preoperative weight loss (HR 2.17, 95 % CI 1.31–3.58) reflecting the advanced stage of disease, microvascular invasion reflecting the aggressive tumor biology (HR 2.03, 95 % CI 1.10–3.76) was the strongest independent predictor for long-term survival.

To summarize, portal vein resection as an add-on in pancreatic resection does not adversely impact immediate, short- and long-term outcome in locally advanced PDAC as preoperative risk factors determine. The anesthesiologic ASA score gives an excellent picture of patient’s preoperative general health conditions not only predicting the anesthesiologic risk alone but also the perioperative risk for pancreatic resections. The feasibility of portal vein resection should therefore be evaluated in every potential candidate at risk.

## Study limitations

Some limitations of the present study have to be acknowledged. Although we could demonstrate that a ASA Score >2 is a strong predictor of postoperative outcome and not the surgical intervention itself, sample size is modest when it comes to random variability, resulting in wide confidence intervals. To limit this potential bias we performed a bootstrap analysis confirming our initial results. However, our findings from a retrospective single-center study will ideally need confirmation in multicenter studies. Second, we modified slightly the definitions of the international study group (ISGPF) for postoperative complications [19] due to the retrospective nature of study design: We have only documented clinically significant pancreatic fistulas; a standardized analysis of drainage liquid for amylase and lipase is not part of our routine protocol. Furthermore, in our cohort, we did not assess the incidence of delayed gastric emptying, as to our institutional protocol, all patients receive a nasogastral tube and prokinetic drugs (metoclopramide and/or domperidone) in the early postoperative period. Residual confounding by patient management at the operating theater and intensive care unit, however, may be still present. Such confounders are impossible to control in an observational retrospective study design. But considering overall death, our main end point, this bias appears to be negligible as patient-related data were retrieved from a validated nationwide Austrian Cancer Database [8].
